# Cancer Screening Recommendations During the COVID-19 Pandemic: Scoping Review

**DOI:** 10.2196/34392

**Published:** 2022-02-24

**Authors:** Sumit K Shah, Pearl A McElfish

**Affiliations:** 1 Office of Community Health and Research University of Arkansas for Medical Sciences Northwest Fayetteville, AR United States; 2 College of Medicine University of Arkansas for Medical Sciences Northwest Fayetteville, AR United States

**Keywords:** COVID-19, cancer prevention and early detection, cancer screenings, breast cancer screening, cervical cancer screening, colorectal cancer screening

## Abstract

**Background:**

Cancer screening tests are recommended to prevent cancer-associated mortality by detecting precancerous and cancerous lesions in early stages. The COVID-19 pandemic disrupted the use of preventive health care services. Although there was an increase in the number of cancer screening tests beginning in late 2020, screenings remained 29% to 36% lower than in the prepandemic era.

**Objective:**

The aim of this review is to assist health care providers in identifying approaches for prioritizing patients and increasing breast, cervical, and colorectal cancer screening during the uncertainty of the COVID-19 pandemic.

**Methods:**

We used the scoping review framework to identify articles on PubMed and EBSCO databases. A total of 403 articles were identified, and 23 articles were selected for this review. The literature review ranged from January 1, 2020, to September 30, 2021.

**Results:**

The articles included two primary categories of recommendations: (1) risk stratification and triage to prioritize screenings and (2) alternative methods to conduct cancer screenings. Risk stratification and triage recommendations focused on prioritizing high-risk patients with an abnormal or suspicious result on the previous screening test, patients in certain age groups and sex, patients with a personal medical or family cancer history, patients that are currently symptomatic, and patients that are predisposed to hereditary cancers and cancer-causing mutations. Other recommended strategies included identifying areas facing the most disparities, creating algorithms and using artificial intelligence to create cancer risk scores, leveraging in-person visits to assess cancer risk, and providing the option of open access screenings where patients can schedule screenings and can be assigned a priority category by health care staff. Some recommended using telemedicine to categorize patients and determine screening eligibility for patients with new complaints. Several articles noted the importance of implementing preventive measures such as COVID-19 screening prior to the procedures, maintaining hygiene measures, and social distancing in waiting rooms. Alternative screening methods that do not require an in-person clinic visit and can effectively screen patients for cancers included mailing self-collection sampling kits for cervical and colorectal cancers, and implementing or expanding mobile screening units.

**Conclusions:**

Although the COVID-19 pandemic had devastating effects on population health globally, it could be an opportunity to adapt and evolve cancer screening methods. Disruption often creates innovation, and focus on alternative methods for cancer screenings may help reach rural and underresourced areas after the pandemic has ended.

## Introduction

Cancer-associated mortality is the second leading cause of death in the United States [1,2]. Cancer screening tests are recommended to prevent cancer-associated mortality by detecting precancerous and cancerous lesions in early stages [3]. The most common routine cancer screenings include breast, colorectal, and cervical [4].

The COVID-19 pandemic disrupted the use of preventive health care services [5]; there was an abrupt decline in cancer screening services throughout 2020 [6]. A report from May 2020 suggested there was a 94% drop in cancer screening tests across the United States, primarily due to disruptions in access to screening tests [7]. Although there was an increase in the number of cancer screening tests beginning in late 2020, screenings remained 29% to 36% lower than in the prepandemic era [8].

The reduction in cancer screenings and other preventative and diagnostic care have been attributed to both health care provider and patient constraints [9-12]. Health care provider constraints included restrictions on elective procedures [9] and a shortage of health care staff due to redeployment to help with pandemic-related care [9,10]. Even when health care providers have increased availability of preventive care and cancer screenings, many patients face constraints. Patient constraints include loss of income and employer-based insurance coverage [11] and fear of contracting COVID-19 during in-person health care visits [12].

The decline in cancer screening resulted in fewer cancer diagnoses in 2020 [13,14] and raises concerns that missed screenings and delayed cancer diagnoses will likely lead to late stage diagnosis and higher cancer-related mortality [7,14]. For example, a study (n=5167) reported a 13.5% (*P*=.03) decrease in colorectal cancer diagnoses during March 2020 to December 2020 compared to the number of patients diagnosed before the pandemic, and the same study showed the average number of stage three colorectal cancer cases (advanced stage cancers) diagnosed per month increased by 68.4% (*P*<.001) [15].

Health care providers must consider ways to increase cancer screening. Therefore, we conducted a scoping literature review to assist health care providers in identifying approaches for prioritizing and increasing cancer screening during the uncertainty of the COVID-19 pandemic. In this review, we focused on the most common cancer screenings: breast, cervical, and colorectal.

## Methods

We used the scoping review framework outlined by Arksey and O’Malley [16] to identify and gather evidence from all sources in the field. The framework is comprised of four stages: (1) identification of relevant literature on multiple databases, (2) screening of identified literature and selection of relevant studies, (3) extraction of data, and (4) summarization and reporting of the findings [16]. The research questions of this review are what methods are recommended for risk stratification and triage of patients for cancer screenings, and what alternative cancer screening methods are recommended?

### Stage 1: Identification of Relevant Literature

The keywords used to identify articles on PubMed and EBSCO databases were “cancer screening and coronavirus,” “cancer screening and COVID-19,” and “cancer screening and SARS-CoV-2.” The articles selected had to include breast, cervical, and colorectal cancer screening. Articles were screened for relevance based on the information provided in the abstract, and those deemed to be relevant by their abstract were fully reviewed. Additional literature was identified from the references of selected articles. A broader search strategy was adopted to include gray literature. These included commentaries and editorials published in peer-reviewed journals, recommendations published by professional organizations or societies, and medical news articles. The literature review ranged from January 1, 2020, to September 30, 2021.

### Stage 2: Screening of Identified Literature and Selection of Relevant Studies

**Figure 1:**
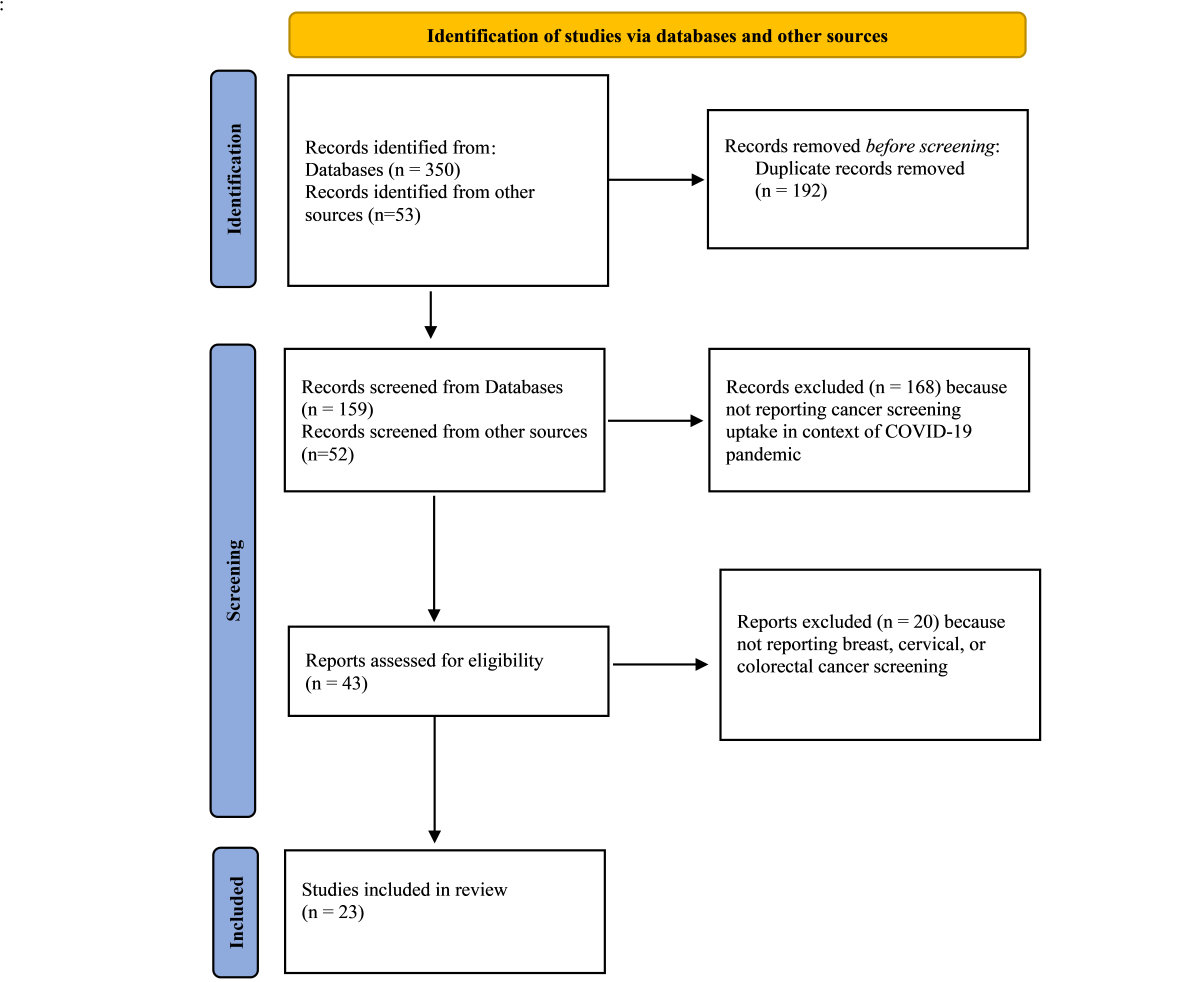
PRISMA (Preferred Reporting Items for Systematic Reviews and Meta-Analyses) flow diagram for new systematic reviews, which includes searches of databases only.

A total of 350 articles were identified from the databases, and an additional 53 articles were identified from references of the relevant articles. After pooling literature from different sources, we found 192 articles were duplicates; duplicates were excluded. Of the remaining 211 articles, 168 were deemed ineligible after screening the abstracts. Of the remaining 43 articles that were fully reviewed, 20 were excluded. Articles not focused on breast, cervical, and colorectal cancer screenings; not suggesting measures to address cancer screening during and after the pandemic; and providing suggestions not substantiated by past literature were excluded. A total of 23 articles were selected for this review. Two authors (SKS and PAM) reviewed the literature and agreed upon the selection of articles. The PRISMA (Preferred Reporting Items for Systematic Reviews and Meta-Analyses) flowchart is provided in Figure 1.

### Stage 3: Extraction of Data

The data points recorded were the article citations, type of article, type of cancer screening discussed, and key recommendations.

### Stage 4: Summarization and Reporting of the Findings

The Results section and tables summarize the data regarding recommendations for risk stratification and triage and alternative cancer screening methods for breast, cervical, and colorectal cancer screenings and report concise information about alternative methods that can be used for cancer screenings.

## Results

The articles included two primary categories of recommendations: (1) risk stratification and triage to prioritize screenings and (2) alternative methods to conduct cancer screenings (Table 1).

**Table 1:** Summary of safely resuming cancer screening services.

Approach	References	Strategies
Risk stratification and triage	Basu et al 2021 [9] Castanon et al 2021 [17] Cohen et al 2020 [18] Corley et al 2021 [19] Croswell et al 2021 [6] Fagundes et al 2021 [20] Gralnek et al 2020 [21] Helsper et al 2020 [22] Houlihan 2020 [23] Isaacs and Leininger 2021 [24] Issaka and Somsouk 2020 [25] Kadakuntla et al 2021 [26] Miller 2021 [27] Orenstein 2020 [28] Pediconi et al 2020 [29] Puricelli Perin et al 2021 [30] Riley 2020 [31] Seguin 2020 [32]	Stratify patients into high-risk, average-risk, and low-risk categories based on age, sex, past medical history, past personal history, or region/area of residence Triage patients based on risk category, prioritizing patients at high risk of cancer, followed by average-risk and low-risk patients
Alternative screening methods	Balzora et al 2020 [33] Castanon et al 2021 [17] Corley et al 2021 [19] Croswell et al 2021 [6] Fagundes et al 2021 [20] Gorin et al 2021 [34] Issaka and Somsouk 2020 [25] Kadakuntla et al 2021 [26] Miller 2021 [27] Miller et al 2021 [35] Orenstein 2020 [28] Ricciardiello et al 2021 [36]	Self-collecting of vaginal or urine samples for cervical cancer screening Self-collection of stool sample for colorectal cancer screening Mobile units outside primary health care facilities for breast cancer screening

### Risk Stratification and Triage

Risk stratification and triage was recommended as an appropriate method for focusing cancer screenings during the COVID-19 pandemic. The recommendation focused on prioritizing those who are most susceptible to developing cancers [6,9,17-32]. Potential criteria considered for categorizing patients into high risk included patients with an abnormal or suspicious result on the previous screening test [27], age group [17,26,32], sex [26], personal medical or family history [18,24,26,27], currently symptomatic or asymptomatic, predisposition to hereditary cancers, and inheritance of cancer-causing mutations [18,26].

Conversely, articles recommended the following patients be deferred until high priority patients are offered cancer screenings: patients with a recent cancer screening with normal results [17,20]; patients who do not have any cancer-related symptoms [18,22]; patients who have taken prophylactic measures such as the human papillomavirus (HPV) prophylactic vaccine [17,20,24]; and patients who do not have medical, personal, or family-related indication for immediate screening [18,19,23,31].

Other recommended strategies included identifying areas facing most disparities [19,34], creating algorithms [24] and using artificial intelligence [28] to create cancer risk scores, leveraging in-person visits to assess cancer risk [6], and providing the option of open access screenings where patients can schedule screenings and can be assigned a priority category by health care staff [26]. Some recommended screening high-risk patients through telemedicine prior to having them come into health care providers [23,29].

In addition to risk stratification and triage, telemedicine was recommended to determine screening for patients with new complaints [18,19,22]. Several articles noted the importance of implementing preventive measures such as COVID-19 screening prior to the procedures [6,9,26], maintaining hygiene measures [19,32], and social distancing in waiting rooms [32].

### Alternative Screening Methods

Several studies discussed using novel and alternative screening methods that do not require an in-person clinic visit and can effectively screen patients for cancers (Table 2). Mailing of self-collection sampling kits was widely suggested as a screening strategy for cervical and colorectal cancers [6,17,19-21,25-28,33-36]. Cervical cancer screening included mailing or pharmacy pickup of kits for self-sampling of vaginal or urine samples that can be tested for HPV strains most likely to cause cancers [17,27,34,35]. Stool-based self-collection kits that are performed at home and mailed for screening were recommended for colorectal cancers [6,19,20,25,26,33-36]. Although self–breast examinations can be done at home, they do not take the place of mammography; therefore, articles recommended implementing and expanding mobile screening units [28,30].

**Table 2:** Alternative approaches to increase cancer screenings.

Cancer type/cancer risk factors	Conventional recommendation/practices	Variation in approaches
Breast cancer	Mammography	Screening at mobile units or small satellite units Follow-up on patients with abnormal results
Cervical cancer	Pap smear Pap smear + HPV^a^ co-testing	Self-collection of vaginal or urine samples at home Follow-up on patients with abnormal results
Colorectal cancer	Colonoscopy Sigmoidoscopy CT^b^ colonography Stool-based tests	Self-collection of stool samples at home Follow-up on patients with abnormal results

^a^HPV: human papillomavirus.

^b^CT: computed tomography.

## Discussion

The number of cancer screenings missed during the COVID-19 pandemic will likely lead to a sharp increase in the number of late-stage cancer diagnoses and increased cancer mortality [14]. As health care providers look for ways to focus their cancer screening efforts, this review provides insights into risk stratification and triage approaches and alternative screening approaches that can be adopted to reduce the impact of COVID-19 on cancer mortality.

Risk stratification and triage approaches focused on prioritizing patients based on personal characteristics, medical history, cancer screening history, and communities facing highest cancer disparities [6,9,17-32]. The literature suggests that older patients at higher risk should be given priority since the risk of cancer increases with age [17,26].

Prioritizing high-risk patients based on the past screening history could help the health care provider prioritize care based on the probability of patients developing cancerous lesions. Several studies have shown that prioritizing high-risk patients based on past medical history is important [6,9,17-32], and studies have reported the effectiveness of the personalized screening approach, demonstrating that the one-size-fits-all approach may not be the best strategy [37-40]. In addition, using algorithms and artificial intelligence to categorize and triage high-risk patients will help navigate large data sets and assist physicians in the decision-making process [24,28].

Alternative cancer screening approaches focused on tests that do not require a clinic or hospital visit can be used to collect samples at home. These alternative methods allow initial screening outside the traditional clinical environment, take fewer clinical resources, and reduce exposure risk to patients. Alternative at-home screening modalities exist for cervical cancer screening [41-43] and colorectal cancer [26]. Studies have evaluated the efficacy of detecting cervical intraepithelial lesions using self-collected samples with samples collected in the doctor’s office and concluded that self-sampling is a safe and effective alternative to screen for cervical cancers [42,43]. Similar to cervical cancer, colorectal cancer screenings can be effectively conducted using noninvasive stool-based test kits at home [44,45]. Studies have shown that stool-based test kits can help reach underresourced communities and increase colorectal cancer screening uptake [46]. Although the stool-based tests have a high false-positive rate [47], patients testing negative can be assured that they do not have colorectal cancers [26].

Follow-up for abnormal results from at-home tests can be provided and help focus limited clinical resources. Although there are not at-home alternatives for mammography, mobile units can provide a way to reach the community [28,30] and reduce exposure risk.

Although the COVID-19 pandemic had devastating effects on population health globally, it could be an opportunity to adapt and evolve our cancer screening recommendations. Disruption often creates innovation, and focus on alternative methods for cancer screenings may help reach rural and underresourced areas after the pandemic has ended.

## Appendix

Multimedia Appendix 1 PRISMA (Preferred Reporting Items for Systematic Reviews and Meta-Analyses) checklist.

## Abbreviations

HPV: human papillomavirus
PRISMA: Preferred Reporting Items for Systematic Reviews and Meta-Analyses
